# Supplementary figures and images for: Genomic Inbreeding and Runs of Homozygosity Analysis of Cashmere Goat
Source: Animals (Basel). 2024 Apr 22;14(8):1246. doi: 10.3390/ani14081246 (PMC11047310; doi:10.3390/ani14081246)

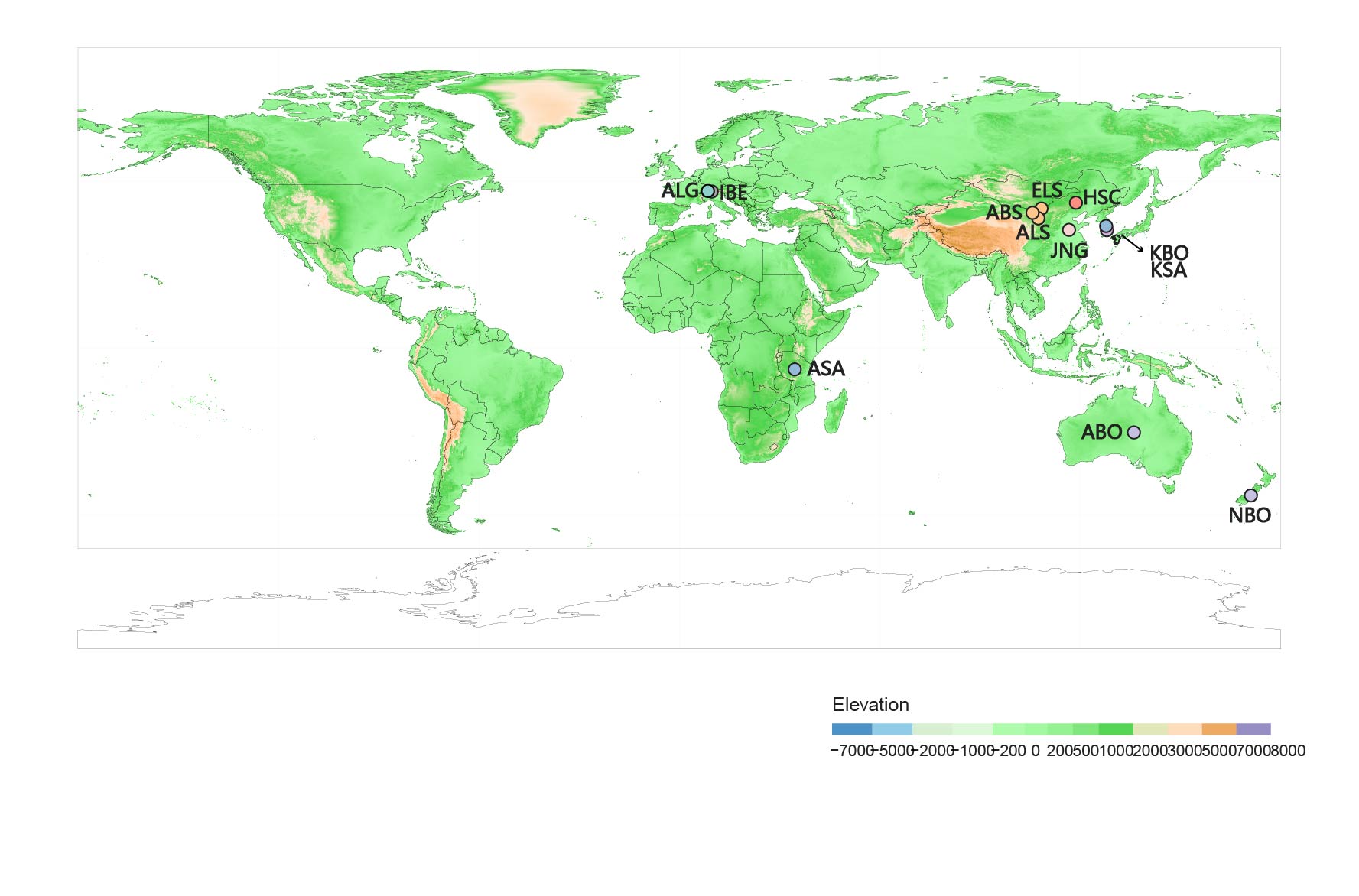

Supplement: Supplementary file 1 [file animals-14-01246-s001.zip › Figure S1.jpg]

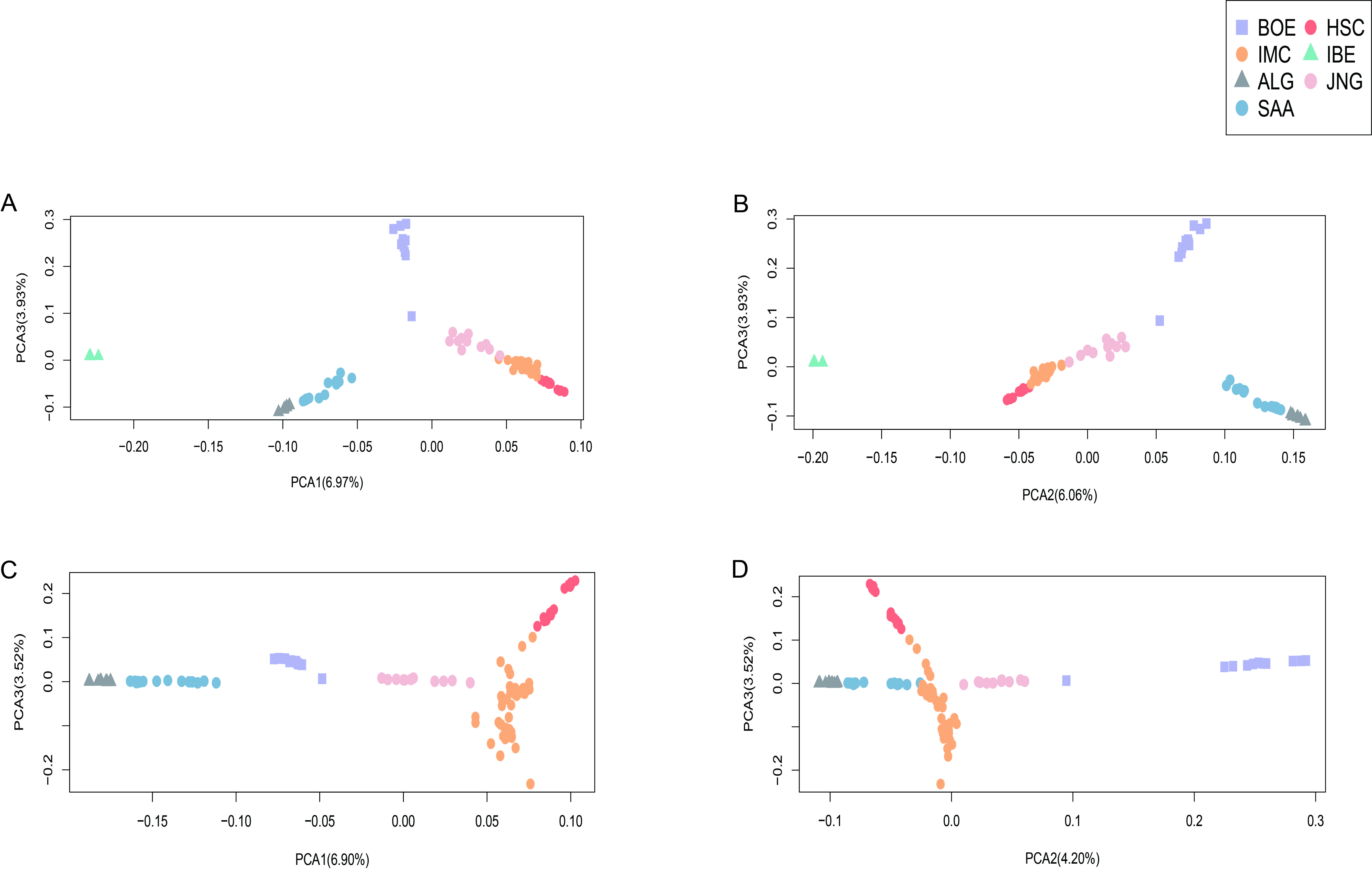

Supplement: Supplementary file 1 [file animals-14-01246-s001.zip › Figure S2.jpg]

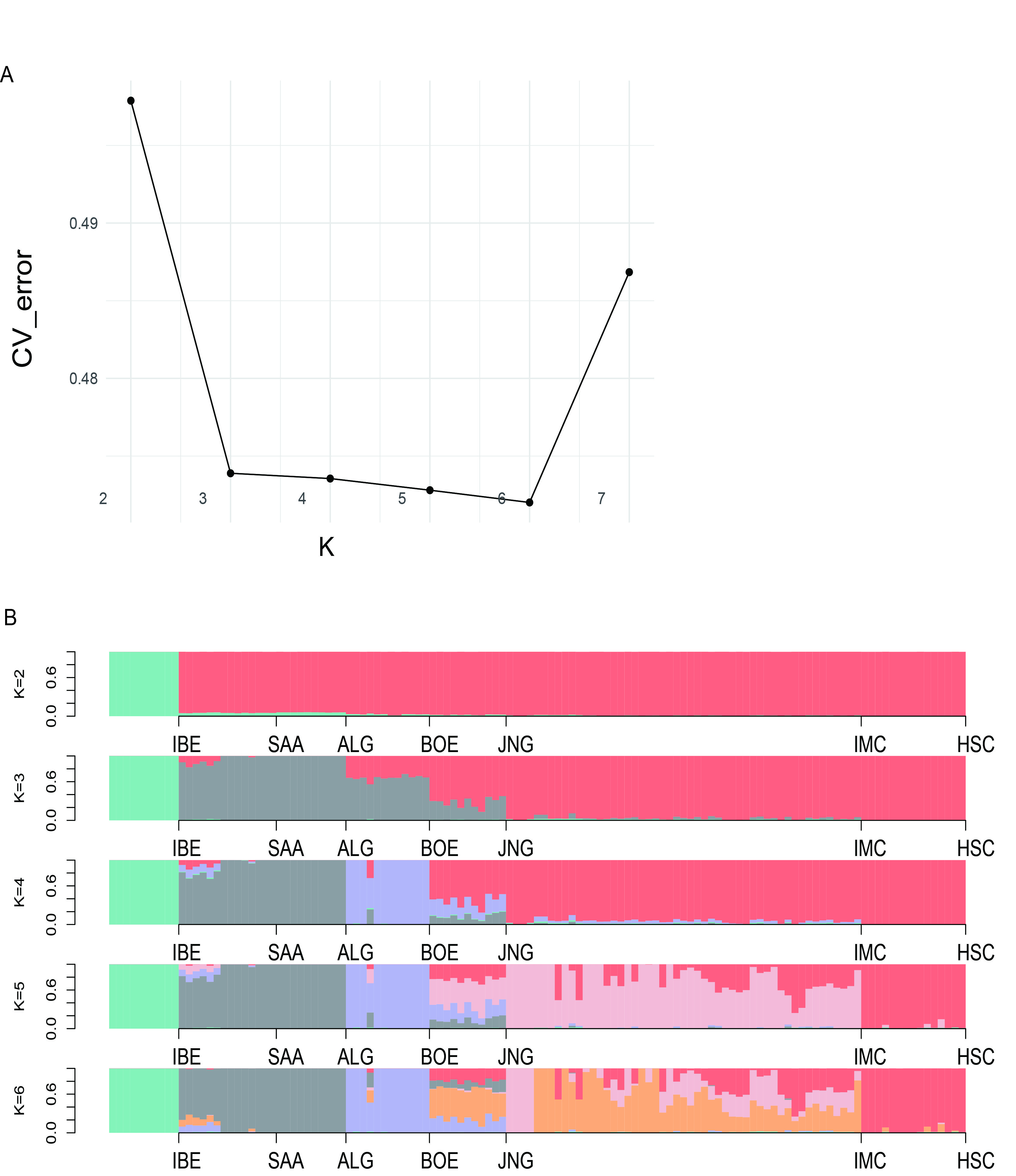

Supplement: Supplementary file 1 [file animals-14-01246-s001.zip › Figure S3.jpg]

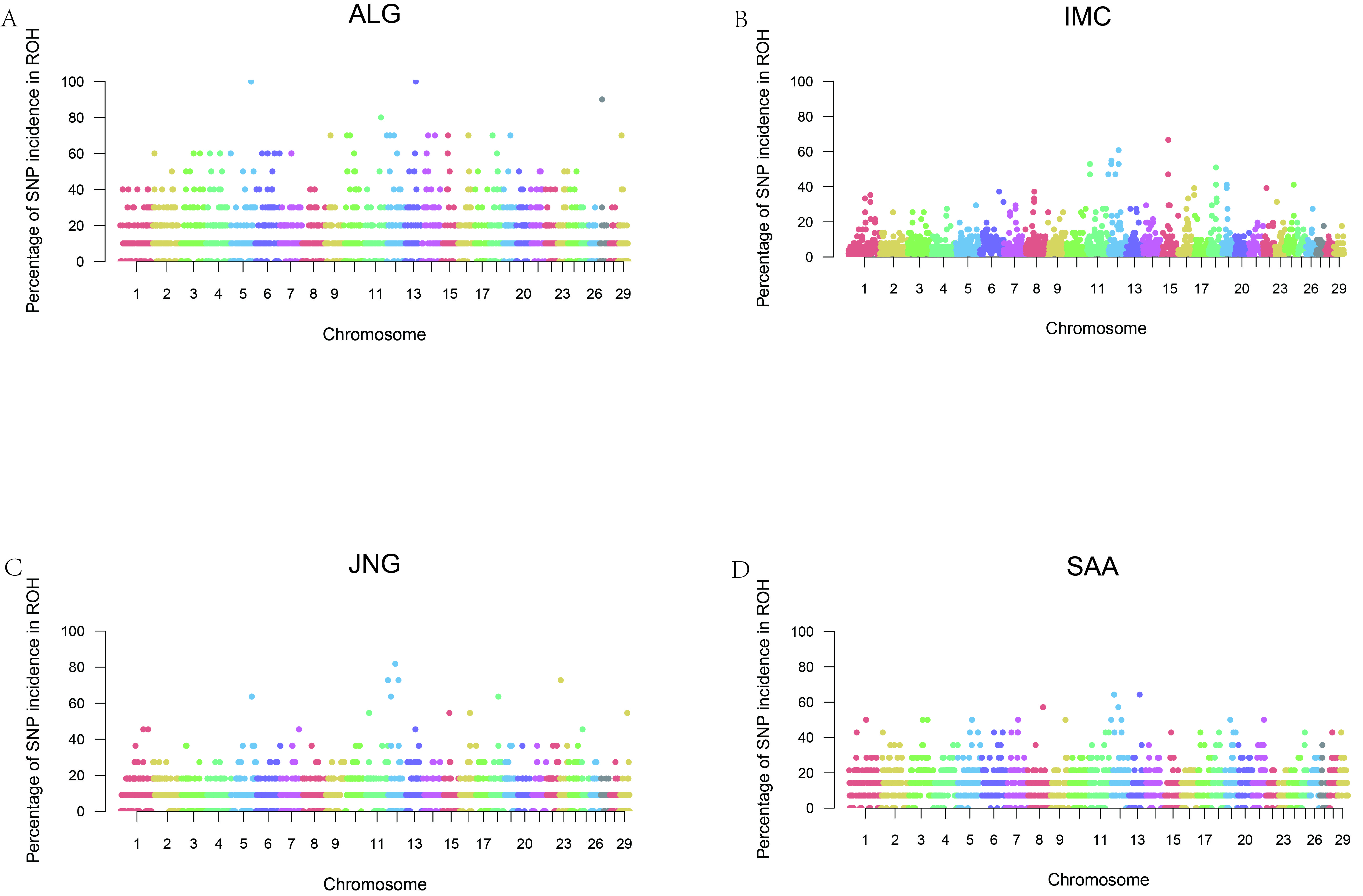

Supplement: Supplementary file 1 [file animals-14-01246-s001.zip › Figure S4.jpg]
